# Transcriptome-wide identification of walnut *PP2C* family genes in response to external stimulus

**DOI:** 10.1186/s12864-022-08856-3

**Published:** 2022-09-08

**Authors:** Chen Sisi, Deng Jieru, Cheng Peidong, Zhang Zhaolong, Wang Yihang, Chen Shuwen, Tang Yan, Wang Tianyu, Yang Guiyan

**Affiliations:** 1 Labortory of Walnut Research Center, College of Forestry, Northwest A & F University, Yangling, 712100 Shaanxi China; 2 Key Laboratory of Economic Plant Resources Development and Utilization in Shaanxi Province, College of Forestry, Northwest A & F University, Yangling, 712100 Shaanxi China

**Keywords:** *Juglans regia*, Protein phosphatase 2C, Bioinformatics, Expression analysis

## Abstract

Walnut is an important economic tree species while confronting with global environmental stress, resulting in decline in quality and yield. Therefore, it is urgent to elucidate the molecular mechanism for the regulation of walnut response to adversity. The protein phosphatase 2C (*PP2C*) gene family participates in cellular processes in eukaryotes through reversible phosphorylation of proteins and signal transduction regulation. However, the stress response function of *PP2C* genes was far to be clarified. Therefore, to understand the stress response mechanism of walnut tree, in this study, a total of 41 *PP2C* genes with complete ORFs were identified from *Juglans regia*, whose basic bio-information and expression patterns in response to multiple stresses and ABA were confirmed. The results showed that the ORFs of *JrPP2C*s were 495 ~ 3231 bp in length, the predicted JrPP2C proteins contained 164 to 1076 amino acids and the molecular weights were 18,581.96 ~ 118,853.34 Da, the pI was 4.55 ~ 9.58. These *JrPP2C* genes were unevenly distributed on 14 chromosomes, among which Chr11 and Chr13 contained the most genes. Phylogenetic analysis found that these JrPP2C proteins were classed into 9 subfamilies, among which group F covered most JrPP2Cs. The JrPP2Cs in the same subfamily exhibited similarities in the composition of conserved domains, amino acid sequences of motifs and exon/intron organization in DNA sequences. Each JrPP2C includes 4 ~ 10 motifs and each motif contained 15 ~ 37 amino acids. Among the motifs, motif1, motif2, motif3 and motif8 were most abundant. Most of the *JrPP2C* genes diversely response to osmotic, cadmium, and *Colletotrichum gloeosporioide* stress as well as ABA treatments, among which *JrPP2C28*, *JrPP2C17*, *JrPP2C09*, *JrPP2C36* were more obvious and deserves further attention. All these results indicated that *JrPP2C* genes play potential vital roles in plant response to multiple stimulus, and are possibly involved in ABA-dependent signaling pathway.

**Supplementary Information:**

The online version contains supplementary material available at 10.1186/s12864-022-08856-3.

## Introduction

Walnut is an important tree species for nut and timber production in the world, and its values of economic, ecological and social have been widely concerned [1]. In China, walnut has a wide range of planting areas and rich varieties. It has gradually become a large-scale agricultural and forestry industry with a wide range of fields, a long industrial chain and an increasingly prominent economic status. It plays an important role in the economic development of the vast mountainous area. However, while the walnut planting area is increasing, it also encounters various problems: the selection of varieties is not necessarily suitable, the yield and quality are unstable, the development of characteristic resources is insufficient, the plantation management is inappropriate and the plants are exposed to various environmental factors (such as drought, high temperature, pests and diseases). These factors restrict the healthy development of the walnut industry. One of the main reasons for these phenomena is that the mechanism of walnut response to adversity is unknown, and walnut cultivation and management measures cannot be effectively formulated. Therefore, in order to provide genetic resources for revealing the stress-resistant response mechanism of walnut and the selection of new germplasm for stress-resistant rootstocks, it is necessary to identify the key genes of walnut in response to stress and then to reveal the stress-resistant regulation mechanism of walnut on this basis.

Reversible phosphates of protein kinases and protein phosphatase-mediated proteins are widely present in organisms and involve in a variety of physiological processes. Protein translation modification can change the physiological and biochemical properties of important functional molecules in the signaling pathway. So it is of great significance for plants to regulate cell cycle, growth and development, hormone and other environmental stimulation [2, 3]. According to the modification function, protein phosphatases (PPs) were divided into three major classes: tyrosine phosphatases (PTPs), serine/threonine phosphatases (PSPs), and dual-specificity phosphatases (DSPTPs) [4]. Among them, PSPs were categorized into three subfamilies—phosphoprotein phosphatases (PPPs), metal-dependent protein phosphatases (PPMs), and aspartate-based phosphatases (APPs). Representative members of the PPP subfamily include PP1, PP2A, PP2B, PP4, PP5, PP6, and PP7. The PPM subclass covered protein phosphatases dependent on manganese/magnesium ions (Mn^2+^/Mg^2+^), such as PP2C and pyruvate dehydrogenase phosphatase [5, 6]. The PP2C subfamily protein is an important branch of the PP family, whose C-terminus has a conserved catalytic domain and the N-terminus is an extension region with different functions; the dephosphorylation of PP2C depends on Mn^2+^ and Mg^2+^ when participating in phosphorylation [7]. The *PP2C* genes are widely present in animals, microorganisms and plants [8], and play regulatory roles in various biological processes. For instance, in mice, *PP2Cβ* played a crucial role during gametogenesis, fertilization, and early stages of embryonic development [9]. In microorganisms, *Ptc6* was believed to be involved in virulence and MAPK signaling in *Fusarium oxysporum* [10]. In plants, *Arabidopsis PP2C49* negatively regulated salt tolerance through inhibition of *AtHKT1;1* [11], wheat *TaPP2C-a10* negatively modified plant drought resistance through ABA signaling [12].

The *PP2C* genes response to plant stress via ABA signaling. ABA receptor PYR/PYL/RCAR in plants receives ABA molecular signals to inhibit protein phosphatase activity, reduce or eliminate the inhibition of *PP2Cs* on downstream kinases (eg, SnRK2s, OST1), and enhance kinase phosphorylation of substrate proteins to participate in plant growth and stress modulation [13, 14]. *Arabidopsis PP2CG1* positively regulates salt stress in an ABA-dependent manner [15]. PeHAB1 could interact with the ABA receptor PYL4 in an ABA-independent manner to reduce tolerance to drought in poplar [16]. In maize, *ZmPP2C26* has a negative regulatory effect against drought stress [17]. Tomato *SlPP2C* gene family that encoding the core component of ABA signaling could regulate tomato fruit development and be induced by drought [18]. Due to the extensive role of *PP2Cs* in plant stress resistance, it has received some attention in recent years. However, it is far to be enough, especial in woody plants, which limits the whole and deep understanding of the target plant in many aspects of life process, such as stomatal switching, growth and development, and stress response. Therefore, in the present study, in order to identify candidate genes for revealing walnut stress response mechanism, walnut *PP2C* genes were selected from *Juglans regia* according to chromosome distribution, gene structure, protein motifs and phylogeny. Meanwhile, five stresses (drought, salt, cadmium, ABA and anthrax) were applied to assess the expression activity of the selected *PP2C* genes. The results of this study will supply new evidence for subsequent study of *JrPP2Cs* respond to stimulus.

## Materials and methods

### Plant materials and treatments

The plant material used in this study was the 3-year-old 'Xiangling' walnut (a phenotype widely grown in China) that grown in a greenhouse (22 ± 2 °C, relative humidity 70 ± 5%, light cycle 14 h light/10 h dark) [19]. 20% (w/v) PEG_6000_, 0.3 mol/L NaCl, and 0.2 mmol/L CdCl_2_ were watered to the roots of the seedlings, respectively, and the leaves were collected at 0 and 6 d and saved in − 80 °C refrigerator for further RNA isolation. For ABA treatment, 30 μmol/L ABA was used and sampling time is 0, 6 and 9 d. For walnut anthracnose treatment, *Colletosporum gloeosporioide* colonies with conidia were rinsed with sterile water and cultivated to the concentration of 10^5^ ~ 10^6^ cells/mL. After the walnut leaves are slightly damaged by friction, spray the prepared anthracnose spore re-suspension on the leaves for treatment and then the leaves were sampled at 0 and 9 d. Each treatment contained 6 seedlings. For tissue expression analysis, the tissues of 6 years old grafted 'Xiangling' including leaves, tender stems, old stems, male flowers, and female flowers were collected on April 13th, 2019, and three biological replicates were applied for each test sample [20].

### Identification of *PP2C* genes in walnut

‘Protein phosphatase’ was used to search for the walnut transcriptomes (sequenced by our research group) under treatments of drought, salt, cadmium, ABA and *C. gloeosporioide*. Then the PP2C protein sequences of *Arabidopsis* were obtained from the TAIR database (https://www.arabidopsis.org/) [21] and used for homology alignment to screen the walnut PP2C members. The ORF Finder (https://www.ncbi.nlm.nih.gov/orffinder/) was used to find the open reading frame (ORF) of potential walnut *PP2C* genes, whose protein sequences were further queried and verified in the NCBI protein database by BLASTp (https://blast.ncbi.nlm.nih.gov/Blast.cgi?PROGRAM=blastp&PAGE_TYPE=BlastSearch&LINK_LOC=blasthome). The molecular weight (MW), theoretical isoelectric point (pI) and amino acid composition were predicted using the ExPASy server (https://web.expasy.org/protparam/). The corresponding gene accession numbers were blast from NCBI. The PP2C domains (PF00481) of all of the walnut were analyzed using HMM (Hidden Markov Model) by searching PFAM (Protein family: http://pfam.sanger.ac.uk/search) and HMMER (https://www.ebi.ac.uk/Tools/hmmer/search/phmmer) [22]. Those proteins lack PP2C domain were removed. NCBI-Conserved domain database (CDD) (https://www.ncbi.nlm.nih.gov/Structure/cdd/wrpsb.cgi) was applied for domain composition analyses.

### Analysis of evolutionary relationship, gene structure and chromosomal locations

To analyze the evolutionary relationship of walnut *PP2C* genes, 78 *Arabidopsis* PP2C protein sequences were download from the TAIR database (https://www.arabidopsis.org/) and 28 *Populus* PP2C protein sequences were obtained from Phytozome v13 (https://phytozome-next.jgi). The PP2C protein sequences of walnut, *Arabidopsis*, and *Populus* trees were compared using the Clustal W2 program [23] and the phylogenetic tree was constructed using the neighbor-joining method in MEGA7 [24]. The evolutionary tree was beautified using the online software itol (https://itol.embl.de/) [25]. JrPP2Cs were classified into subgroups according to the topology of the phylogenetic tree and referring to the previous studies on *A. thaliana* [26].

The gene structure map of the exon-intron of *JrPP2C*s was determined by Gene Structure Display Server 2.0 (GSDS 2.0: http://gsds.gao-lab.org/). The MEME online tools (http://alternate.meme-suite.org/) were used to analyze the conservative motifs with the following parameters: the number of motifs is 12, allowing any number repetitions, motif width is from 15 to 36. The chromosomal location information of 41 JrPP2Cs in the walnut genome was confirmed from NCBI (https://www.ncbi.nlm.nih.gov/), the walnut genome data refer to *J. regia* (assembly Walnut 2.0) [27–29]. The motif domain and chromosomal location were visualized by TBtools [30].

### Expression analysis of *JrPP2Cs*

The total RNA of all samples were extracted by CTAB (cetyltrimethylammonium ammonium bromide) method [31]. The RNA concentration was determined and reverse-transcribed to cDNA by PrimeScript™ RT reagent Kit (CWBIO, Beijing, China) after treated by DNA digestion enzyme. The cDNA was diluted 10 times and used as the template of quantitative real-time PCR (qRT-PCR). QRT-PCR was performed using the SYBR Green Real time PCR Master mix (CWBIO) with an internal reference gene of walnut *18S rRNA* (HE574850) [32]. The primers used are shown in Table S1. The instrument used for the quantitative reaction is the StepOne™ Real Time PCR system produced by Applied Biosystems. The reaction procedures were: 94 °C for 30 s, 45 cycles of 94 °C for 12 s, 60 °C for 45 s, 72 °C for 45 s; 81 °C for 1 s, 3 replicates per sample. The quantitative results were analyzed by 2^-ΔΔCT^ method [33]. The data were analyzed using the SPSS package (SPSS, Chicago, Illinois, USA). Sample variability is expressed as standard deviation. Expression differences between different time points and 0 d were analyzed by T test (*P* < 0.05). The results were visualized in Tbtools software and Origin 2017 and the results are represented using heat maps [30].

## Results

### Sequence characteristics and chromosomal locations of walnut* PP2C* genes

**Fig. 1:**
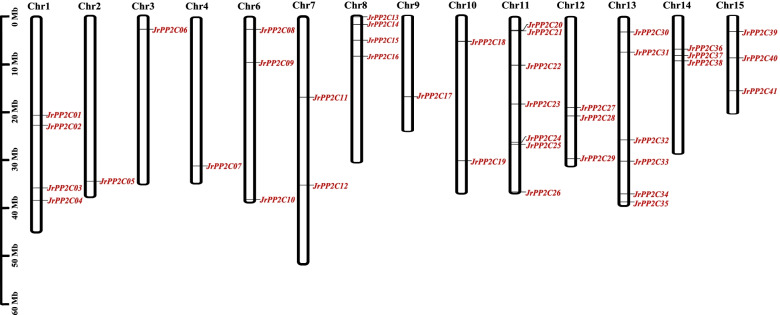
Chromosome distribution of walnut *PP2C* genes. Chromosome localization is based on the physical location (Mb) of 15 walnut chromosomes. Chromosome numbers are displayed at the top of each bar chart. Locations of walnut *PP2C* genes in chromosomes were obtained from the Walnut 2.0 (https://www.ncbi.nlm.nih.gov/assembly/GCF_001411555.2/). Scale bar on the left indicated the length (Mb) of walnut chromosomes

A total of 44 putative *JrPP2C* genes were screened from walnut transcriptome, among these 44, 3 lacked PP2C catalytic domain confirmed by PFAM and SMART tools. Therefore, 41 genes in *J. regia* were identified as *PP2C* family members. These 41 *PP2C* genes were anchored to corresponding chromosomes and designated as *JrPP2C1* to *JrPP2C41* according to their order on the chromosomes (Table 1, Fig. 1). The ORFs of 41 *JrPP2C* genes were 495 ~ 3231 bp in length, the molecular weights of the deduced peptides were 18,581.96 ~ 118,853.34 Da with 164 ~ 1076 amino acids, and the theoretical isoelectric point (pI) was 4.55 ~ 9.58 (Table 1).

**Table 1:** Information of the *PP2C* gene family in *J. regia*

Gene names	Accession No.	Gen ID	Chromosome	Number of amino acids/aa	Molecular weight/Da	Theoretical pI	ORF
*JrPP2C01*	XP_018813464.1	LOC108985575	Chr1	471	52,127.43	5.26	1416
*JrPP2C02*	XP_018814869.1	LOC108986641	Chr1	300	32,877.41	6.67	903
*JrPP2C03*	XP_035542087.1	LOC109008983	Chr1	324	35,486.41	6.42	975
*JrPP2C04*	XP_018814919.1	LOC108986673	Chr1	1076	118,853.34	5.13	3231
*JrPP2C05*	XP_018850794.1	LOC109013230	Chr2	283	31,395.71	6.14	852
*JrPP2C06*	XP_018848910.1	LOC109011952	Chr3	194	22,241.75	9.58	585
*JrPP2C07*	XP_018856771.1	LOC109019011	Chr4	678	75,176.6	5.85	2037
*JrPP2C08*	XP_035546983.1	LOC108981366	Chr6	329	35,483.44	4.8	990
*JrPP2C09*	XP_018816109.1	LOC108987629	Chr6	428	46,707.05	5.33	1287
*JrPP2C10*	XP_018808952.1	LOC108982116	Chr6	371	41,053.52	5.47	1116
*JrPP2C11*	XP_018814254.2	LOC108986182	Chr7	386	42,257.33	5.27	1161
*JrPP2C12*	XP_018826078.1	LOC108995052	Chr7	423	46,711.7	5.11	1272
*JrPP2C13*	XP_035549139.1	LOC109000954	Chr8	236	26,210.93	8.99	711
*JrPP2C14*	XP_018809272.1	LOC108982376	Chr8	378	41,029.39	5.38	1137
*JrPP2C15*	XP_041021790.1	LOC109007388	Chr8	283	31,009.1	6.79	852
*JrPP2C16*	XP_035549051.1	LOC109022244	Chr8	385	42,096.86	5.17	1158
*JrPP2C17*	XP_018842619.1	LOC109007408	Chr9	818	89,856.47	5.2	2457
*JrPP2C18*	XP_035550417.1	LOC108988842	Chr10	415	45,537.27	5.55	1248
*JrPP2C19*	XP_018821199.1	LOC108991415	Chr10	268	28,923.27	4.55	807
*JrPP2C20*	XP_018839661.1	LOC109005271	Chr11	369	41,037.52	5.97	1110
*JrPP2C21*	XP_018837177.1	LOC109003487	Chr11	427	45,944.73	7.92	1284
*JrPP2C22*	XP_018829600.1	LOC108997697	Chr11	397	44,139.37	8.62	1194
*JrPP2C23*	XP_018810588.1	LOC108983394	Chr11	687	75,934.92	5.54	2064
*JrPP2C24*	XP_018846910.1	LOC109010509	Chr11	351	38,711.74	4.62	1056
*JrPP2C25*	XP_018843134.1	LOC109007763	Chr11	902	101,265.8	5.87	2709
*JrPP2C26*	XP_018833633.1	LOC109005189	Chr11	292	31,504.85	4.98	879
*JrPP2C27*	XP_018832233.1	LOC108999793	Chr12	164	18,581.96	4.99	495
*JrPP2C28*	XP_018825718.1	LOC108994803	Chr12	526	57,118.28	4.93	1581
*JrPP2C29*	XP_041000376.1	LOC109003005	Chr12	389	42,901.11	6.92	1170
*JrPP2C30*	XP_018820545.1	LOC108990886	Chr13	281	30,862.86	8.95	846
*JrPP2C31*	XP_018829268.1	LOC108997438	Chr13	374	40,927.7	5.16	1125
*JrPP2C32*	XP_018821783.2	LOC108991840	Chr13	428	46,952.52	8.83	1287
*JrPP2C33*	XP_018824697.1	LOC108994067	Chr13	389	42,690.62	5.94	1170
*JrPP2C34*	XP_018817932.1	LOC108988961	Chr13	431	46,431.09	8.69	1296
*JrPP2C35*	XP_018809730.1	LOC108982727	Chr13	350	38,920.51	5.57	1053
*JrPP2C36*	XP_018835605.1	LOC109002354	Chr14	387	41,763.87	6.11	1164
*JrPP2C37*	XP_018830588.1	LOC108998487	Chr14	288	31,829.22	6.96	867
*JrPP2C38*	XP_018823298.1	LOC108992999	Chr14	727	80,848.43	5.55	2184
*JrPP2C39*	XP_018844698.1	LOC109008885	Chr15	546	58,544.61	4.79	1641
*JrPP2C40*	XP_018815014.1	LOC108986745	Chr15	534	58,234.96	5.43	1605
*JrPP2C41*	XP_018854698.1	LOC109016775	Chr15	326	35,196.77	7.72	981

41 *JrPP2C* genes were dispersed on 14 chromosomes. On each chromosome, the number of *JrPP2C* varies drastically, ranging from 1 to 7, the largest number of *JrPP2C* members was observed on chromosomes 11 with 7 genes, followed by chromosome 13 with 6 genes, whereas the least numbers were revealed on chromosomes 2, 3, 4 and 9, each contains only 1 gene (Fig. 1), suggesting the uneven distribution of *JrPP2C* genes on chromosomes.

### Phylogenesis and classification of JrPP2C proteins

**Fig. 2:**
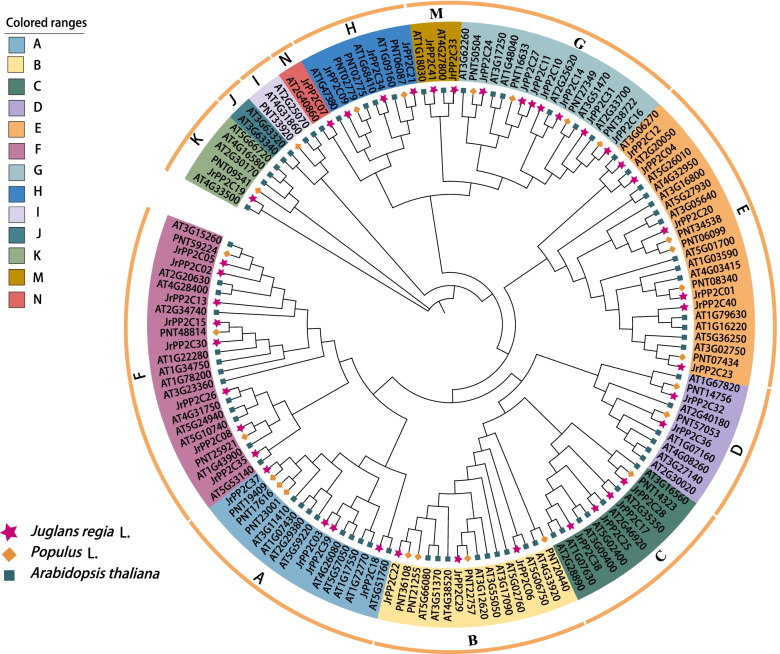
Phylogenetic relationship of JrPP2C proteins. Alignments of 145 PP2C domains from *J. regia*, *Populus*, and *Arabidopsis* were performed with Clustal W. MEGA7 was used to construct a phylogenetic tree with the Neighbor-Joining method. Different colors indicate different subfamily members according to sequence similarity annotation analysis

To investigate the phylogenetic relationships of PP2C proteins between walnut and other plants, an un-rooted phylogenetic tree was constructed based on the alignments of PP2C domains from walnut, *Arabidopsis* and *Populus* using the Neighbor-Joining method. According to the classification of *Arabidopsis* [34], the PP2Cs of these three plants were divided into eleven groups: Group A and C each includes 4 JrPP2Cs, they were JrPP2C03, JrPP2C18, JrPP2C37, JrPP2C39, and JrPP2C17, JrPP2C25, JrPP2C28, JrPP2C38, accordingly; Group B and H contains 3 JrPP2Cs, they were JrPP2C06, JrPP2C22, JrPP2C29, and JrPP2C09, JrPP2C21, JrPP2C34, accordingly; Groups D and M covers 2 members, they were JrPP2C32, JrPP2C36, and JrPP2C33, JrPP2C41, respectively; Group K and N each only included 1 JrPP2Cs, they were JrPP2C19 and JrPP2C07; JrPP2C01, JrPP2C04, JrPP2C12, JrPP2C20, JrPP2C23, and JrPP2C40 were classed into group E; JrPP2C10, JrPP2C11, JrPP2C14, JrPP2C16, JrPP2C24, JrPP2C27 and JrPP2C31 were grouped in G class; The other 8 (JrPP2C02, JrPP2C05, JrPP2C08, JrPP2C13, JrPP2C15, JrPP2C26, JrPP2C30, JrPP2C35) belongs to group F (Fig. 2).

### The conserved motif composition and domain of JrPP2Cs

**Fig. 3:**
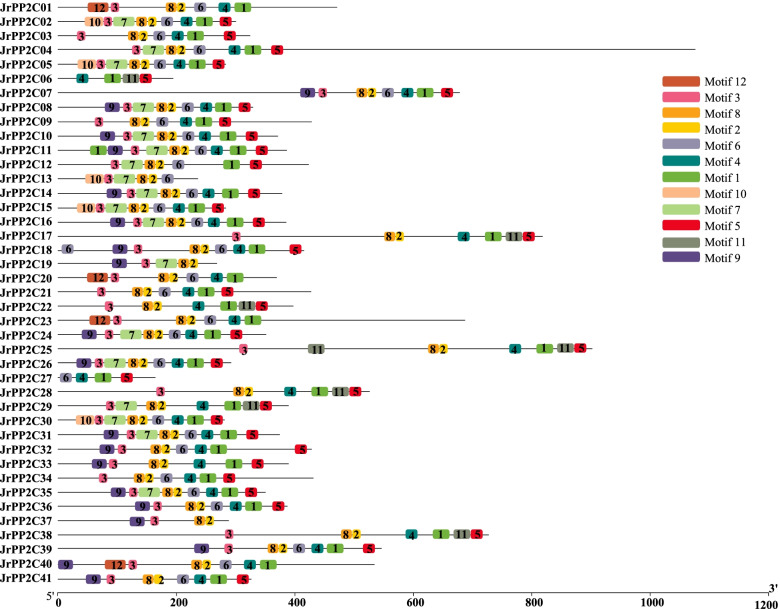
The conserved motifs of JrPP2C proteins. The 12 conserved motifs are represented with different color boxes, and the motif sequence logos are displayed in the upper right corner. The dark line shows the length of proteins

**Fig. 4:**
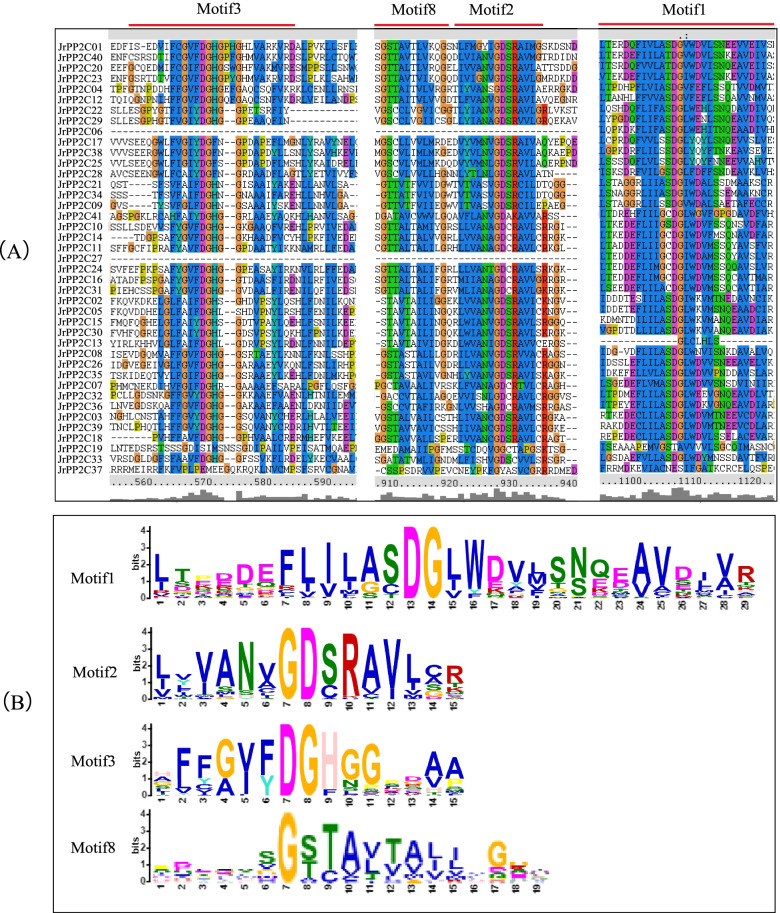
Multiple sequence alignment of the motifs of 41 JrPP2C proteins (**A**) and the sequence logo of motif1, motif2, motif3 and motif8 (**B**)

A total of 12 conserved motifs were detected from 41 JrPP2C proteins using MEME tool [35], each motif contains 15 ~ 36 amino acids (Table 2), and each sequence includes 4 ~ 10 motifs (Fig. 3). The most frequent motifs of JrPP2Cs are motif1, motif2, motif3, and motif8, whose amino acid sequences are highly conserved, and they represent the PP2C domain (Fig. 4). Among 41 JrPP2Cs, JrPP2C11 has most motifs (total 10 — two motif1, motif2, motif3, motif4, motif5, motif6, motif7, motif8, motif9); JrPP2C06, JrPP2C27 and JrPP2C37 were the genes that containing the least (only four) motifs. JrPP2C02, JrPP2C05, JrPP2C15, and JrPP2C30 shared 9 same motifs, they are motif10, motif3, motif7, motif8, motif2, motif6, motif4, motif1, and motif5. JrPP2C08, JrPP2C10, JrPP2C14, JrPP2C16, JrPP2C24, JrPP2C26, JrPP2C31, and JrPP2C35 also shared 8 same motifs and only one (motif9) is different from the motifs (motif10) in JrPP2C02, JrPP2C05, JrPP2C15, and JrPP2C30. Total 9 JrPP2Cs contain 8 motifs, among them JrPP2C07, JrPP2C32, JrPP2C36, JrPP2C39, JrPP2C41 have 8 identical motifs (motif9, motif3, motif8, motif2, motif6, motif4, motif1, motif5). There are 13 genes with 7 motifs, of which JrPP2C17, JrPP2C22, JrPP2C28, and JrPP2C38 have the same 7 motifs (motif3, motif8, motif2, motif4, motif1, motif11, motif5); JrPP2C20, JrPP2C01, and JrPP2C23 shared same motifs (motif12, motif3, motif8, motif2, motif6, motif4, motif1); JrPP2C09, JrPP2C21, and JrPP2C34 contain other 7 same motifs (motif3, motif8, motif2, motif6, motif4, motif1, motif5).

**Table 2:** Motif sequences of JrPP2C proteins identified by MEME tool

Motif	Width	Best possible match
Motif1	29	LTPDDEFLILASDGLWDVLSNZEAVDJVR
Motif2	15	LVVANVGDSRAVLCR
Motif3	15	AFFGVFDGHGGPDAA
Motif4	21	LAVSRAFGDWYLKKPVVSEPP
Motif5	21	LVEEALRRGSKDBITVIVVDL
Motif6	21	DHKPERSDERERIEAAGGRVS
Motif7	37	YLKEHLFENJLKDPDFWTDTEKAIRSAYRQTDAAFLK
Motif8	19	PDLASSGSTAVTAJIVGGT
Motif9	26	VRSGSASDIGRREYMEDEHIIIPDLL
Motif10	32	ITHGFHLVKGKSNHPMEDYVVAEFKQFKGHE
Motif11	29	PEGDPARHLVEELLFRAAKKRGMDYHELL
Motif12	36	GRIFLNGASKIASJFTQQGKKGTNQDAMIVWENFGS

**Fig. 5:**
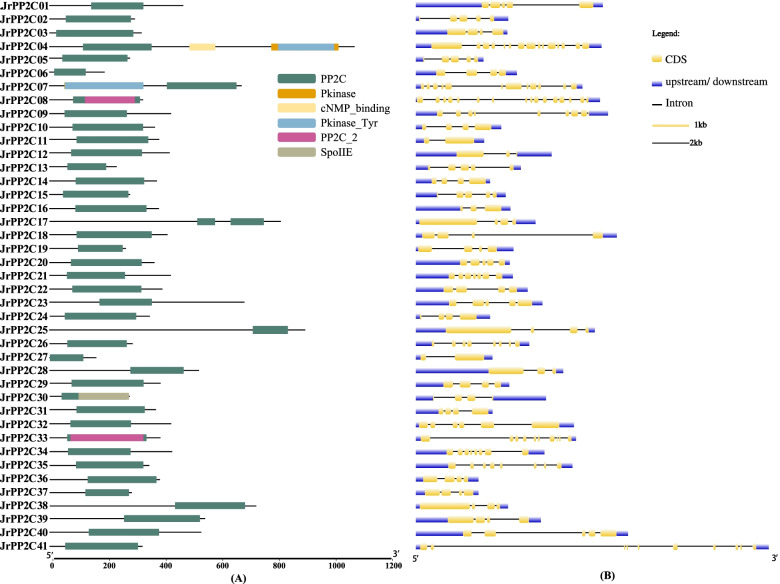
Protein domain and gene structure of *JrPP2C* genes. **A** Distribution of conserved domains within JrPP2C proteins. The relative positions of each domain are shown in color boxes, the names were indicated on the right. **B** Exon/intron structure of *JrPP2C* genes. Yellow boxes represent exons, gray lines represent introns and blue boxes represent untranslated regions. The sizes of genes can be estimated by the scale at the bottom

In addition, PFAM analysis showed that 41 JrPP2C proteins covered various conserved domains or segmental duplications of PP2C domain (Fig. 5A, Table S2). All JrPP2C proteins had similar PP2C domain and one or more other structures (Pkinas, cNMP_binding, Pkinase_Tyr, PP2C_2, SpoiIE), for instance, JrPP2C04, JrPP2C07. JrPP2C08 and JrPP2C33 are similar sharing the PP2C_2 domain (Fig. 5A). As to the segmental duplications of the domains, all JrPP2Cs have domains of PP2Cc (accession No.: cd00143, smart00332), PP2C (accession No.: pfam00481) and PTC1 (accession No.: COG0631), and most have five different intervals to display the domain hits. JrPP2C04 is the most one covering 20 intervals. JrPP2C07 have the second most number of domains with 14. JrPP2C01 covers only 5 intervals, the one with the fewest number of domains, and the remaining 38 JrPP2Cs all have 6 domains (Table S2). These structural similarities and differences suggest that JrPP2Cs may have functional overlap and specificity.

### Gene structure of *JrPP2Cs*

Exon–intron structural diversity within a gene family is an important clue for the evolutionary and functional analyses. To know the components of the *JrPP2C* gene structure, the exons and introns, including their amount and distribution among *JrPP2C* genes were examined. The results revealed that most members in the same subfamily shared similar exon numbers and different exon and intron lengths. The number of introns and exons of these 41 *JrPP2C*s ranges from 1 to 15, and 2 to 16, respectively (Fig. 5B). In detail, *JrPP2C08* contains 15 introns and 16 exons, the largest number. Secondly, *JrPP2C04* contains 14 introns and 15 exons, *JrPP2C07* has 11 introns, 12 exons; *JrPP2C41* and *JrPP2C33* contains 9 introns and 10 exons, while *JrPP2C11* and *JrPP2C27* both contain only 1 intron and 2 exons, the least number. In addition, many genes have the same number of introns and exons, in detail, *JrPP2C26*, *JrPP2C34*, and *JrPP2C35* each contains 7 introns and 8 exons, *JrPP2C01*, *JrPP2C02*, *JrPP2C05*, *JrPP2C13*, *JrPP2C15*, *JrPP2C20*, *JrPP2C23*, and *JrPP2C40* each contains 4 introns and 5 exons. *JrPP2C03*, *JrPP2C06*, *JrPP2C10*, *JrPP2C17*, *JrPP2C18*, *JrPP2C19*, *JrPP2C22*, *JrPP2C24*, *JrPP2C25*, *JrPP2C29*, *JrPP2C30*, *JrPP2C31*, *JrPP2C36*, *JrPP2C37*, *JrPP2C38* and *JrPP2C39*, each contains 3 introns and 4 exons (Fig. 5B).

### Tissue expression specificity of *JrPP2Cs*

**Fig. 6:**
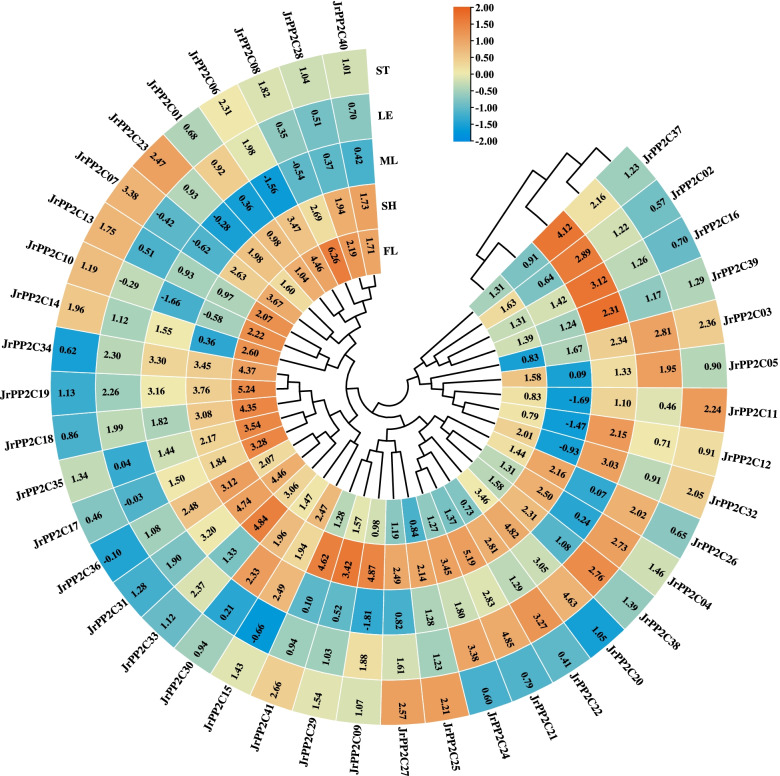
Expression patterns of *JrPP2C* genes in five walnut tissues. Heatmap of *JrPP2C* expression data was created by Tbtools. ML, male flowers; ST, old stems (stems of 2 years old and older branches); SH, tender stems (stems of the new shoots in the current year); FL, female flowers; LE, leaves, respectively. Heat map is presented in blue/yellow/orange colors that indicate low/medium/high expression

To investigate the potential role of *JrPP2C*s, female flowers (FL), male flowers (ML), old stems (ST, stems of 2 years old and older branches), tender stems (SH, stems of the new shoots in the current year) and leaves (LE) were collected and the transcription level of 41 *JrPP2C* genes were confirmed using qRT-PCR method. The results showed that most *JrPP2C*s were expressed in all tissues with various profiles and could group into following types (Fig. 6).

①Gene expression levels were highest in FL among the five tissues, containing *JrPP2C01*, *JrPP2C06*, *JrPP2C07*, *JrPP2C08*, *JrPP2C10*, *JrPP2C13*, *JrPP2C14*, *JrPP2C17*, *JrPP2C18*, *JrPP2C19*, *JrPP2C28*, *JrPP2C34*, and *JrPP2C35*. Among these members, *JrPP2C08* showed highest expression level (6.26) while *JrPP2C01* was the lowest one (1.04).

②Gene expression levels were highest in SH among the five tissues, including *JrPP2C09*, *JrPP2C20*, *JrPP2C21*, *JrPP2C24*, *JrPP2C26*, *JrPP2C29*, *JrPP2C31*, *JrPP2C33*, *JrPP2C36*, *JrPP2C40* and *JrPP2C41.* Among which *JrPP2C21* displayed the highest expression level (5.19) while *JrPP2C40* was the lowest one (1.73).

③Gene expression levels were highest in ML among the five tissues, covering *JrPP2C02*, *JrPP2C12*, *JrPP2C15*, *JrPP2C16*, *JrPP2C30*, *JrPP2C32*, *JrPP2C37*, and *JrPP2C39.* Among them, *JrPP2C37* was transcribed to a maximum value (4.12) while *JrPP2C12* was the minimum one (2.15).

④Gene expression levels were highest in LE among the five tissues, consisting of *JrPP2C03* (2.81), *JrPP2C04* (2.73), *JrPP2C05* (1.95), *JrPP2C22* (3.27), and *JrPP2C38* (2.76)*.*

⑤Gene expression levels were highest in ST among the five tissues, grouped with *JrPP2C11* (2.24), *JrPP2C23* (2.47), *JrPP2C25*(2.21), and *JrPP2C27* (2.57)*.*

### Expression activity of *JrPP2Cs* to biotic and abiotic stresses as well as ABA treatment

**Fig. 7:**
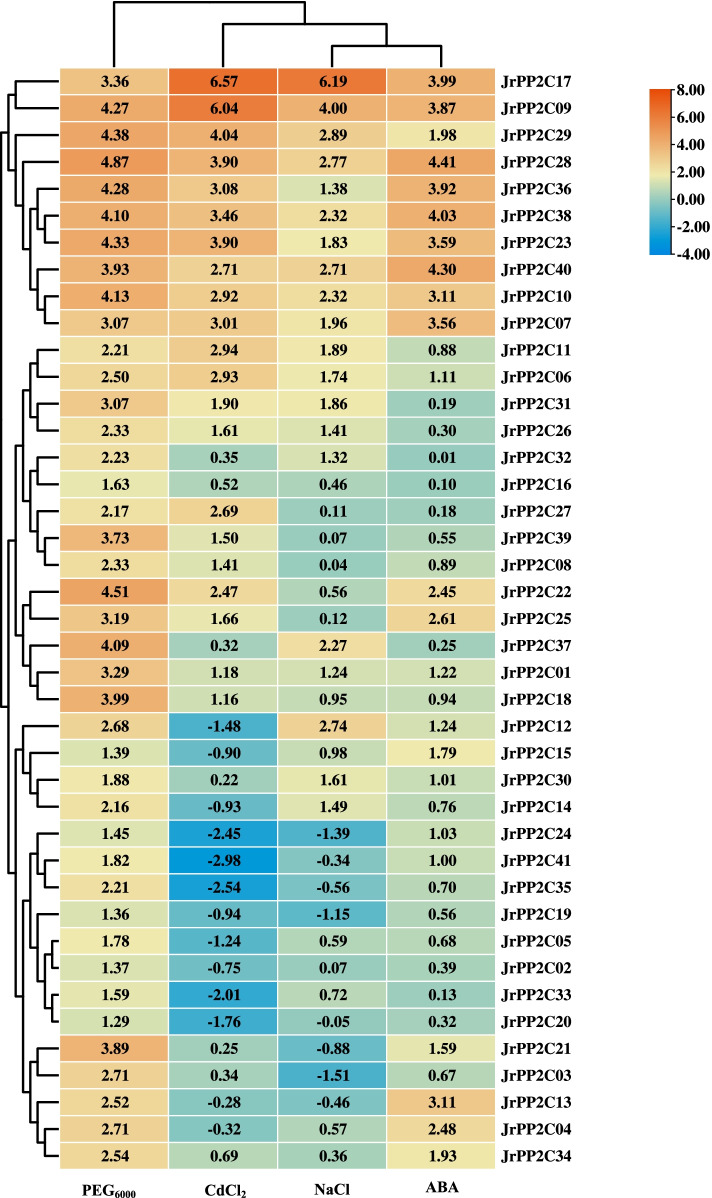
Expression patterns of *JrPP2Cs* under abiotic stress conditions at 6 days. Four experimental stress conditions are denoted as 20% (w/v) PEG_6000_, 0.3 mol/L NaCl, 0.2 mmol/L CdCl_2_, and 30 μmol/L ABA to 3-year-old 'Xiangling' walnut seedlings. The expression is relative to the expression of the internal reference gene and at 0 day. Heatmap of *JrPP2C* expression data was created by Tbtools. Row clustering was applied. Heatmap is presented in blue/yellow/orange colors that indicate low/medium/high expression

**Fig. 8:**
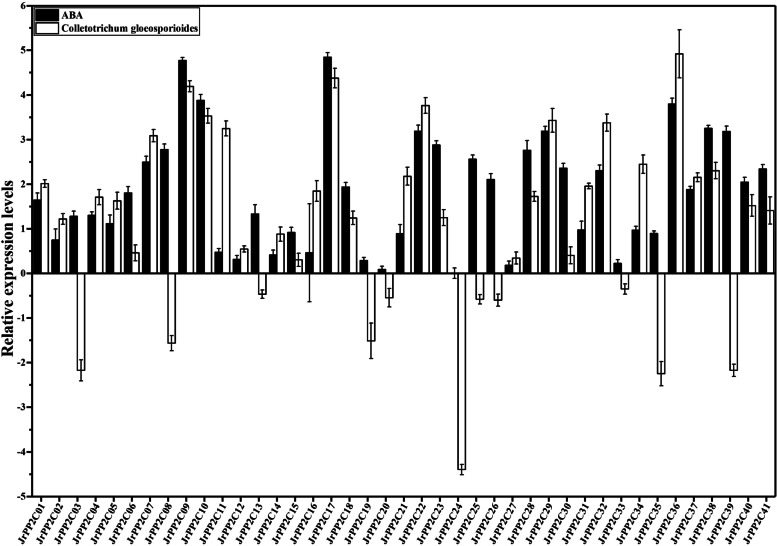
Expression patterns of *JrPP2Cs* under ABA and *Colletotrichum gloeosporioides* stress at 9 days. 10^5^–10^6^ cells/mL *Colletosporum gloeosporioide* spore re-suspension was incubated to the walnut leaves for anthracnose stress. ABA concentration is 30 μmol/L. The expression is relative to the expression of the internal reference gene and at 0 day

To explore the potential function of *JrPP2Cs* in response to common stresses and whether involving in ABA signalling, the expression of 41 *JrPP2Cs* were analyzed under stresses of drought, salt, heavy metal, and *C. gloeosporioides* as well as treatment of ABA (Figs. 7, 8 and S1).

#### Under drought stress

The expression of these 41 *JrPP2Cs* were showed the same trend under PEG_6000_ stress. After 6 d of PEG_6000_ stress, their relative expression was increased, and the average expression value was 2.86. The transcription of nine genes (*JrPP2C28*, *JrPP2C22*, *JrPP2C29*, *JrPP2C23*, *JrPP2C36*, *JrPP2C09*, *JrPP2C10*, *JrPP2C38*, *JrPP2C37*) exceeded 4.00, among them *JrPP2C28* displayed the highest induction (4.87). The relative expression level of 10 genes (*JrPP2C30*, *JrPP2C41*, *JrPP2C05*, *JrPP2C16*, *JrPP2C33*, *JrPP2C24*, *JrPP2C15*, *JrPP2C02*, *JrPP2C19*, *JrPP2C20*) were less than 2.00. In which, *JrPP2C20* was the one that induced with lowest expression level, the transcription of *JrPP2C28* is 3.78-fold of *JrPP2C20* (Fig. 7), suggesting that *JrPP2C28* may be the most potential candidate gene for walnut drought stress regulation in these 41 *JrPP2Cs*.

#### Under salt stress

The expression of 41 *JrPP2Cs* under NaCl stress could class to three groups: (i) Genes with relative expression levels greater than 1 that covered 20 genes (*JrPP2C17*, *JrPP2C09*, *JrPP2C29*, *JrPP2C28*, *JrPP2C12*, *JrPP2C40*, *JrPP2C10*, *JrPP2C38*, *JrPP2C37*, *JrPP2C07*, *JrPP2C11*, *JrPP2C31*, *JrPP2C23*, *JrPP2C06*, *JrPP2C30*, *JrPP2C14*, *JrPP2C26*, *JrPP2C36*, *JrPP2C32*, *JrPP2C01*), and the average relative expression of these 20 genes was 2.30, of which *JrPP2C17* (6.19) was the most prominent, followed by *JrPP2C09* (4.00). (ii) Genes with relative expression levels ranging from 0 to 1 containing 13 genes (*JrPP2C15*, *JrPP2C18*, *JrPP2C33*, *JrPP2C05*, *JrPP2C04*, *JrPP2C22*, *JrPP2C16*, *JrPP2C34*, *JrPP2C25*, *JrPP2C27*, *JrPP2C02*, *JrPP2C39*, *JrPP2C08*), their mean value of relative expression is 0.43, which was only 19% of the average value of the above group. (iii) Genes with relative expression levels less than 0. The expression of *JrPP2C20*, *JrPP2C41*, *JrPP2C13*, *JrPP2C35*, *JrPP2C21*, *JrPP2C19*, *JrPP2C24*, and *JrPP2C03* were suppressed by NaCl stress, in which the suppressed most obviously genes were *JrPP2C19* (− 1.15), *JrPP2C24* (− 1.39), and *JrPP2C03* (− 1.51) (Fig. 7), indicating that *JrPP2C17* may be a salt stress response gene that the worthiest one for further study.

#### Under heavy metal stress

Under the treatment of CdCl_2_, the relative expression levels of 41 *JrPP2Cs* genes changed obviously, 68% of the genes was up-regulated, of which *JrPP2C17* (6.57) was induced to the highest level, *JrPP2C09* (6.04) was ranked at the second site, followed by *JrPP2C29* (4.04). Others were transcribed lower than 4.00. The expression level of *JrPP2C23*, *JrPP2C28*, *JrPP2C38*, *JrPP2C36*, *JrPP2C07*, *JrPP2C11*, *JrPP2C06*, *JrPP2C10*, *JrPP2C40*, *JrPP2C27*, *JrPP2C22* was range from 2.00 to 4.00, and their average level is 3.09. *JrPP2C13*, *JrPP2C04*, *JrPP2C02*, *JrPP2C15*, *JrPP2C14*, and *JrPP2C19* were all suppressed by CdCl_2_ stress, the expression value of *JrPP2C33*, *JrPP2C24*, *JrPP2C35*, and *JrPP2C41* were − 2.01, − 2.45, − 2.54, and − 2.98, respectively. Except for the above genes, the expression of other 14 genes (*JrPP2C31*, *JrPP2C25*, *JrPP2C26*, *JrPP2C39*, *JrPP2C08*, *JrPP2C01*, *JrPP2C18*, *JrPP2C34*, *JrPP2C16*, *JrPP2C32*, *JrPP2C03*, *JrPP2C37*, *JrPP2C21*, *JrPP2C30*) varied little under Cd stress, and the values were between 0 and 1 (Fig. 7). These results tell us that *JrPP2C17* may be also the Cd response candidate.

#### Expression to *C. gloeosporioides* stress

73% of the 41 *JrPP2C* genes were up-regulated and other 27% were down-regulated under *C. gloeosporioides* stress. All genes can be classified into three groups based on their relative expression levels: (i) Relative expression levels greater than 1, including 15 genes (*JrPP2C34*, *JrPP2C38*, *JrPP2C21*, *JrPP2C37*, *JrPP2C01*, *JrPP2C31*, *JrPP2C16*, *JrPP2C28*, *JrPP2C04*, *JrPP2C05*, *JrPP2C40*, *JrPP2C41*, *JrPP2C23*, *JrPP2C18*, *JrPP2C02*). Among them, the expression level of 8 genes (*JrPP2C36*, *JrPP2C17*, *JrPP2C09*, *JrPP2C22*, *JrPP2C10*, *JrPP2C29*, *JrPP2C32*, *JrPP2C11*) were greater than 3. *JrPP2C36* had the highest expression and the value is 4.92. (ii) Genes with relative expression levels in the range of 0 ~ 1. *JrPP2C14*, *JrPP2C12*, *JrPP2C06*, *JrPP2C30*, *JrPP2C27*, *JrPP2C15* were in this group with a mean relative expression level of 0.49. (iii) Genes with relative expression levels less than 0. The expression of *JrPP2C33*, *JrPP2C13*, *JrPP2C20*, JrPP2C25, *JrPP2C26*, *JrPP2C19*, *JrPP2C08*, *JrPP2C03*, *JrPP2C39*, *JrPP2C35*, *JrPP2C24* were all suppressed by *C. gloeosporioide* stress, *JrPP2C06*, *JrPP2C30*, *JrPP2C27*, and *JrPP2C15* were down-regulated to a level below − 1, whose mean value was − 2.34. Notably, *JrPP2C24* was suppressed most obviously, and the value is − 4.40 (Fig. 8). These results suggested that if we want to understand the molecular mechanism of walnut resistance to *C. gloeosporioides*, *JrPP2C36* is an important candidate gene.

#### Under ABA treatment

All *JrPP2Cs* were up-regulated by ABA with varied expression profiles that could be classified into two categories: (i) Genes whose peak relative expression levels appeared at 6 d, including *JrPP2C23*, *JrPP2C07*, *JrPP2C28*, *JrPP2C40*, *JrPP2C38*, *JrPP2C36*, *JrPP2C25*, *JrPP2C21*, *JrPP2C34*, *JrPP2C15*, *JrPP2C13*, *JrPP2C04*, *JrPP2C20*, *JrPP2C19*, *JrPP2C11*, *JrPP2C14*, *JrPP2C12*, *JrPP2C24*. Among them, *JrPP2C28*, *JrPP2C40*, and *JrPP2C38* were induced to a level exceed 4.00. (ii) Genes those induced by ABA to maximum level at 9 h contained the genes apart those in subgroup (i) and *JrPP2C27*. In sub-family ii, the top two genes in expression level were *JrPP2C17* (4.85) and *JrPP2C09* (4.77); then *JrPP2C10*, *JrPP2C36*, *JrPP2C29*, *JrPP2C22*, and *JrPP2C39* were also up-regulated to a level higher than 3.00. While *JrPP2C16*, *JrPP2C20*, *JrPP2C27*, and *JrPP2C33* were the genes with little change at 6 and 9 d and their expression were close to 0 (Figs. 7, 8 and S1), implying the varied relation between the *JrPP2Cs* and ABA.

## Discussions

The *PP2C* gene family is one of the largest families of plant and has been identified as important members playing crucial roles in phytohormone signaling, developmental processes, biotic and abiotic stress responses [8, 17, 36], however, *PP2C* genes from walnut trees was still not reported. In order to reveal the adversity adaptation mechanism of walnuts then to provide a basis for walnut cultivation and management to ensure the yield and quality, in this study, 41 walnut *PP2C* genes those may have potential functions in stress response were identified (Table 1). The sequence characteristics (ORF length, amino acid number, molecular weight, and pI) of *JrPP2C*s (Table 1) were ranged similarly as other species, for instance, the molecular weights of PP2C proteins from *Pyrus bretschneideri*, tomoto and current walnut were 7.5 ~ 243 [37], 6.7 ~ 120 [26], and 18.6 ~ 119 kDa, respectively. In terms of evolutionary relationship, the 41 JrPP2C proteins shared a high similarity with the members of PP2C family of *Arabidopsis* and poplar, and could be classified into eleven subfamilies with reference to the classification in *Arabidopsis* [38], and wild soybean [39] (Fig. 2). Meanwhile, except the PP2C conserved domain, PP2C proteins usually contain other domains which might bind potential functional sites thereby activating their function [40]. JrPP2C proteins in this study all have PP2C domain as well as other one or more conserved domains (Pkinas, cNMP_binding, Pkinase_Tyr, PP2C_2, SpoiIE) with differential domain segment duplications (Fig. 5A and Table S2). Multi-sequence comparisons show that 41 JrPP2Cs are highly conserved, and most JrPP2Cs included motif1, motif2, motif3, motif8 (Fig. 3, Table 2), in which motif3 (AFFGVFDGHGGPDAA) presumed to be a marker of PPM phosphatase [8], confirming that these 41 JrPP2Cs belong to PP2C protein family and shared potential varied functions.

Gene structure is also a cue for functions. The 41 *JrPP2C* genes were located in different chromosomes at different sites (Fig. 1) with changeable numbers and distributions of exon and intron (Fig. 5B). In organisms, exons perform phenotypic regulation by encoding protein regions throughout the organism’s genome, so the length and location of exons contain important biological information. The loss/gain of intron position and length is slow, so intron positions can often retain information about gene homology [41]. Many studies of exon/intron structure have shown that most members in the same subfamily have similar exon numbers and different exon and intron lengths [42, 43]. In this study, we found that the number of exons/introns in group A, B, and C was exactly equal, and most of the gene structures in group F, G, and H were similar, while some genes (such as *JrPP2C04*, *JrPP2C07*, and *JrPP2C41*) are quite different from other genes (such as *JrPP2C37*, *JrPP2C19*, and *JrPP2C25*) (Figs. 2, 5B), indicating the functional similarity and specificity of these *JrPP2C* genes. Moreover, the gene and protein structural features of *JrPP2Cs* were similar to those of *PP2C* in *Glycine max* [44], *Gossypium hirsutum* [45], *Brassica rapa* [46] and *Brachypodium distachyon* [47]. Soybean *PP2Cs* could control plant growth and development [44]. Cotton *PP2C* gene family plays critical role in organ and fiber development, as well as abiotic stress tolerance [45]. *BraPP2Cs* has been demonstrated potential ability to regulate biotic and abiotic stress, and *BdPP2CA6* was involved in ABA and stress signaling pathways [34]. Therefore, we speculate these *JrPP2C* genes may relate to the life activity and adversity response of walnut.

To understand whether these *JrPP2C*s are involved in growth and development or tissue expression specificity, the transcription levels of 41 *JrPP2C*s were detected in various tissues, and the results showed that all *JrPP2C*s displayed strong expression in leaves, tender stems, old stems, male flowers, and female flowers (Fig. 6). This observation was similar to the expression pattern of other gene families in walnut and *PP2C*s in other species. For instance, five *MYB* genes could express in a varied pattern in walnut leaves, tender stems, old stems, male flowers, and female flowers and believed to be important candidates for walnut breeding [20]. *JrWRKY2* and *JrWRKY7* displayed obvious expression level in walnut pistil, terminal leaf, other leaves and stems, implying the potential involvement in metabolic processes leading to nut formation [48]. Most wheat *TaPP2C* genes exhibited a wide range of transcription in leaf, stem, root, spike, and grain tissues those related to different developmental stages [8]. 29 *B. rapa PP2C* paralogous gene pairs were detected from various tissues (root, stem, leaf, flower, and silique) [46]. According to the current results and other reports on *PP2C* genes, we believe that *JrPP2C*s genes are correlated with walnut growth and development. Meanwhile, *JrPP2C08* has the highest tissue expression activity (Fig. 6), therefore, it may have the most research potential in the regulation of walnut tissue development.

Considering that the adversity of drought, salt stress, heavy metal pollution and diseases as well as pathogens will affect the growth and yield of walnut, to confirm whether *JrPP2Cs* might be related to the stress response of walnut, the transcriptional activities of 41 *JrPP2C* genes were analyzed under abiotic stress (PEG_6000_, NaCl, CdCl_2_) and biotic stress (*C. gloeosporioide*). The results showed that all *JrPP2Cs* could response to above stresses with various degrees, the relative expression levels of 11 genes were increased under above stresses, among which *JrPP2C09* and *JrPP2C17* were induced more obviously than other genes, especially in response to NaCl and CdCl_2_ stress. Under drought stress, all *JrPP2C* genes were induced. In response to *C. gloeosporioide* stress, the most obvious induction was *JrPP2C36* and *JrPP2C17* (Figs. 7, 8, S1), implying the potential different response ability of these *JrPP2C*s to specific adversity, and may play vital and wide role in drought response. Gene expression is an important and basic way for gene function prediction, for example, *RsHSFs* were judged to play a crucial role in the biological process of salt stress response by analyzing the relatively high expression levels of *RsHSF*-*11* and *RsHSF*-*22* [49]. *JrWRKY2* and *JrWRKY7* were found to be induced by drought, salt and cold, which were further confirmed as drought tolerance regulators [48]. Therefore, we believe that *JrPP2Cs* are important candidate genes of walnut in response to drought, salt, Cd and anthracnose, and the genes with large changes in expression activity deserve further attention.

Protein phosphatases alter protein function by removing phosphate groups from phosphorylated proteins. Studies have shown that ABA plays an important role in plant protein phosphorylation and that some *PP2Cs* are involved in plant stress regulation through the ABA pathway [36, 50, 51], and that ABA receptors (PYR/PYL/RCAR: pyrabactine resistance/PYR-like/regulatory components of ABA response) receive ABA signals and selectively interact with evolved branch A PP2Cs and regulate downstream SnRK2s-type kinases, which in turn regulate the expression of other transcription factors through multiple phosphorylations in response to various stresses [52, 53]. To clarify whether the response of *JrPP2Cs* to adversity was related to ABA, walnut was treated with ABA for the same duration as each adversity treatment (6 and 9 d), and the expression of each *JrPP2C* was analyzed and found that all *JrPP2C* genes could be induced to different degrees after treatment with ABA (Figs. 7, 8, S1). Moreover, the genes that were significantly up-regulated by ABA were also significantly up-regulated by above stresses. For example, *JrPP2C28*, *JrPP2C40*, *JrPP2C38*, *JrPP2C17*, *JrPP2C36*, and *JrPP2C09*, which had higher relative expression levels under ABA for 6 d, were up-regulated more obviously by PEG_6000_, NaCl, and CdCl_2;_*JrPP2C28* even had the highest relative expression levels under both drought and ABA treatments. *JrPP2C19* and *JrPP2C20* were transcribed lowly under PEG_6000_, NaCl, CdCl_2_ as well as ABA treatment for 6 d. *JrPP2C36*, *JrPP2C17*, *JrPP2C09*, *JrPP2C22*, *JrPP2C10*, *JrPP2C29*, whose expression levels were prominent under *C. gloeosporioide* stress, also showed higher expression levels at 9 d of ABA treatment (Fig. 8). It can be seen that the involvement of walnut *PP2C* in stress regulation correlated with ABA. This is similar to other reported ABA-related genes. For example, *JrWRKY2* was induced to a similar expression patter under ABA and drought stress, further, *JrWRKY2* was believed to regulate *JrGSTU23* and *JrVHAc4* in plant drought tolerance via ABA signal pathway [19, 48]. *JrVHAG1* was induced by CdCl_2_ and further confirmed that its Cd-responsive function is also achieved through the ABA signal pathway [54]. *PbrPP2C10*, *PbrPP2C11*, *PbrPP2C15*, and *PbrPP2C18* were up-regulated by exogenous ABA, as a presumption that *PbrPP2C* is related to ABA [37]. Therefore, based on the performance of *JrPP2Cs* under different stress and ABA in this study and other previous reports, we believe that the response of walnut *PP2C* family genes to stress is related to ABA signal.

Moreover, the *PP2C* family has many members with different functions achieved by various ways. For example, *BdPP2CA6* positively regulates salt tolerance in transgenic *Arabidopsis* via interacting with *BdPYLs* and *BdSnRK2* [47]. The *SlPP2C* gene contributes to tomato resistance to bacterial blight and may be regulated by many light-response elements in the promoter region [26]. *Betula platyphylla BpPP2C1* regulates salt stress tolerance involving in ABA signaling pathway, flavonoid biosynthetic pathway, reactive oxygen species (ROS) metabolism, oxidative stress and anion transport [7]. Cold-response elements were found in the promoter region of 31 *Broussonetia papyrifera BpPP2C*s; *Bp01g0320* was found to act as a hub protein; *Bp01g0512* and *Bp09g1278* played key roles relating to ABA-signaling and MAPK cascades, respectively [55]. *ZmPP2C*-*A10* gene negative regulated maize response to drought stress linking endoplasmic reticulum (ER) stress signaling [56]. In the current study, based on basic biological information, tissue expression and expression analysis under different stresses, the potential functions of the walnut *JrPP2C* family genes were clarified, and several potential members (*JrPP2C09*, *JrPP2C28*, *JrPP2C17*, *JrPP2C36*) were identified. In the follow-up research on walnut stress resistance and characteristic germplasm breeding, we will combine the above possible pathways (such as interaction, upstream regulatory elements, ABA signaling, flavonoid biosynthetic pathway).

## Conclusions

A total of 41 *JrPP2C* genes were identified and their distribution on chromosomes, gene structure, conserved motif, and evolutionary relationships were analyzed. The results show that *JrPP2Cs* are highly conserved, and the protein structure contains special sequences of the PP2C family. *JrPP2Cs* could express in most tissues, and *JrPP2C08* is transcript most obviously. Under the stresses of drought, salt, heavy metals, ABA and anthrax bacteria, the relative expression of most walnut *JrPP2C* genes changed significantly, among which *JrPP2C09*, *JrPP2C28*, *JrPP2C17*, and *JrPP2C36* were relatively obvious, which deserve further attention and research, and these results implyed that walnut *JrPP2C* genes may resist drought, salt stress, heavy metals, and anthrax. This study provides useful information for further study of the function and response mechanism of the *JrPP2C* genes.

## Supplementary Information

**Additional file 1: Table S1.** The primers used in the study.

**Additional file 2: Table S2.** The list of PP2Cs domain hits.

**Additional file 3: Fig. S1.** Expression patterns of *JrPP2Cs* under ABA treatment (30 μmol/L) at 6 and 9 days. Heatmap of *JrPP2C* expression data was created by Tbtools. Row clustering was applied. Heatmap is presented in blue/yellow/orange colors that indicate low/medium/high expression. The expression is relative to the expression of the internal reference gene and at 0 day.

## Data Availability

All the data were presented in the main manuscript and additional supporting files. The Arabidopsis related datasets generated and/or analysed during the current study are available in the TAIR database (https://www.arabidopsis.org/).
