# Supplementary material for: Transcriptome-wide identification of walnut PP2C family genes in response to external stimulus
Source: BMC Genomics. 2022 Sep 8;23:640. doi: 10.1186/s12864-022-08856-3 (PMC9461273; doi:10.1186/s12864-022-08856-3)
Supplement: Supplementary file 1 — Additional file 1: Table S1. The primers used in the study. [file 12864_2022_8856_MOESM1_ESM.docx]

Table S1. The primers used in the study

| Primer name | Forward primer | Reverse primer |
| --- | --- | --- |
| JrPP2C01-F/R | 5′-AGTACTTGCAGTAGCAGG-3′ | 5′-CAATCATGGCATCCTGGT-3′ |
| JrPP2C02-F/R | 5′-TGTGTGCTGATGACTTGC-3′ | 5′-CATCATGACCTGAATGGC-3′ |
| JrPP2C03-F/R | 5′-TTGCCATGAACGTCTCCA-3′ | 5′-AACCAAGGCAGTAGAACC-3′ |
| JrPP2C04-F/R | 5′-CGGTTCAACCAATTGGAC-3′ | 5′-GAACACTGAGCTCCGAAT-3′ |
| JrPP2C05-F/R | 5′-GTTAGGTTCGTCAGATCC-3′ | 5′-TTCTGTCCAGAAGTCAGG-3′ |
| JrPP2C06-F/R | 5′-ACAAGAACTCAGGTCCAG-3′ | 5′-TCAGATGTCAGCACAGGT-3′ |
| JrPP2C07-F/R | 5′-ATCAGAGGTTGCTGTACC-3′ | 5′-CCGGATGATCTAGCATAC-3′ |
| JrPP2C08-F/R | 5′-GATCATAGTGTCCTCCAG-3′ | 5′-TTCGGCAGTTCTTGAACC-3′ |
| JrPP2C09-F/R | 5′-TGGATCTGCTGCTGCTAT-3′ | 5′-CAGCAGGTTCAAGTATGC-3′ |
| JrPP2C10-F/R | 5′-TGTGTGTGAAGGATGCAG-3′ | 5′-CAGATGTGAGTATCCTCC-3′ |
| JrPP2C11-F/R | 5′-ATGCTGGACGTGAAGTAC-3′ | 5′-GTGTTCATCCTCCATGGA-3′ |
| JrPP2C12-F/R | 5′-ACCCACAGTCATCAGAT-3′ | 5′-CCTTGAATCTGTGTCCT-3′ |
| JrPP2C13-F/R | 5′-AAGGCATCAGCTTCCTT-3′ | 5′-ACACGATCTCCTAGGTG-3′ |
| JrPP2C14-F/R | 5′-TCATGAAGACGAAGACC-3′ | 5′-CATCCTCGACAATGAAC-3′ |
| JrPP2C15-F/R | 5′-CTACTCTCAGCTTGTAGG-3′ | 5′-GATGTTCCTGTAGGTAGG-3′ |
| JrPP2C16-F/R | 5′-CTGTGGAAGATCAGACCT-3′ | 5′-AGAAGGGAAGTCTGCAGT-3′ |
| JrPP2C17-F/R | 5′-TCCACTCCAAGGACAGTT-3′ | 5′-ACGTATGCATCACCAAGC-3′ |
| JrPP2C18-F/R | 5′-TAGATCTCCTGGAGCCTT-3′ | 5′-CCTTTCCCTACACAATGC-3′ |
| JrPP2C19-F/R | 5′-ACTCAACACGGAAGACTC-3′ | 5′-GACATGAACCCTGGTATG-3′ |
| JrPP2C20-F/R | 5′-TCCTCCATGTTCAATGGG-3′ | 5′-TCATGTCTTCTTGGCACC-3′ |
| JrPP2C21-F/R | 5′-GAGCTTAGGAATGAGAAGG-3′ | 5′-TCCTTGAGGAATTGCACTC-3′ |
| JrPP2C22-F/R | 5′-CTGGTCAGCATATGAGTG-3′ | 5′-CTCTGAGGTGAACCTCTT-3′ |
| JrPP2C23-F/R | 5′-GCTTCATAGGATTCCTGG-3′ | 5′-ATGTTCACTTCCCAGTGG-3′ |
| JrPP2C24-F/R | 5′-TGCCTGTGTTGGAGGATA-3′ | 5′-ATGCTTCAGGTCCTCCAT-3′ |
| JrPP2C25-F/R | 5′-TCGAGCACCCTTGATTCT-3′ | 5′-AGCAATGAGGAACAGGCT-3′ |
| JrPP2C26-F/R | 5′-AGTGCTCTCACCTTCAAC-3′ | 5′-GCATCAGCTATGGCTGAT-3′ |
| JrPP2C27-F/R | 5′-GAGGAGTTAGGTGGATAC-3′ | 5′-AGACTGACTGCATACTGG-3′ |
| JrPP2C28-F/R | 5′-GGCTATCAATGCAATGGC-3′ | 5′-TGACATGGTGCTCTGAAG-3′ |
| JrPP2C29-F/R | 5′-AGGACAGCATGTGAATGG-3′ | 5′-TCTCTGACGTGAACTCTG-3′ |
| JrPP2C30-F/R | 5′-CCATGGAGGATTACCATG-3′ | 5′-CCAAGTCAGGATTGTGAG-3′ |
| JrPP2C31-F/R | 5′-CCAACCCAATGAGAGATG-3′ | 5′-ACAGATGTGCTCATCCTC-3′ |
| JrPP2C32-F/R | 5′-TCCCAAGTGTCTTGCAAG-3′ | 5′-GTTCTCCATCATCTCAAG-3′ |
| JrPP2C33-F/R | 5′-TACCAAGTCCTCAGTTGC-3′ | 5′-TCCCTGAGGAACTTGACT-3′ |
| JrPP2C34-F/R | 5′-TGCTGCATTGATCAGCAG-3′ | 5′-CATAGCACCCAACACATG-3′ |
| JrPP2C35-F/R | 5′-GGTTGATACAAGTGCTCC-3′ | 5′-AATACTCAGCTGCACGAG-3′ |
| JrPP2C36-F/R | 5′-GAAGCGATAACCCTAACC-3′ | 5′-TCTTCCTCCAGCACTTC-3′ |
| JrPP2C37-F/R | 5′-GAGTGAGAACCAAGCTTC-3′ | 5′-AAGCTGAGTTCTCCACAG-3′ |
| JrPP2C38-F/R | 5′-CCTTCTGCTACGTAAGAC-3′ | 5′-CATCAGATTCCTCGGGAT-3′ |
| JrPP2C39-F/R | 5′-CCACTTAGACGTCACTAG-3′ | 5′-CTGTAACGTCATCAGCAG-3′ |
| JrPP2C40-F/R | 5′-CTGCAGCATCAATGTCTTC-3′ | 5′-CTTGGCAACCATATGACC-3′ |
| JrPP2C41-F/R | 5′-ATGAGTGAAGGCACCCATT-3′ | 5′-CCATGTCCATCATAGATTGC-3′ |
| 18S rRNA-F/R | 5'-GGTCAATCTTCTCGTTCCCTT-3' | 5'-TCGCATTTCGCTACGTTCTT-3' |
