# Supplementary figures and images for: Transcriptome-wide identification of walnut PP2C family genes in response to external stimulus
Source: BMC Genomics. 2022 Sep 8;23:640. doi: 10.1186/s12864-022-08856-3 (PMC9461273; doi:10.1186/s12864-022-08856-3)

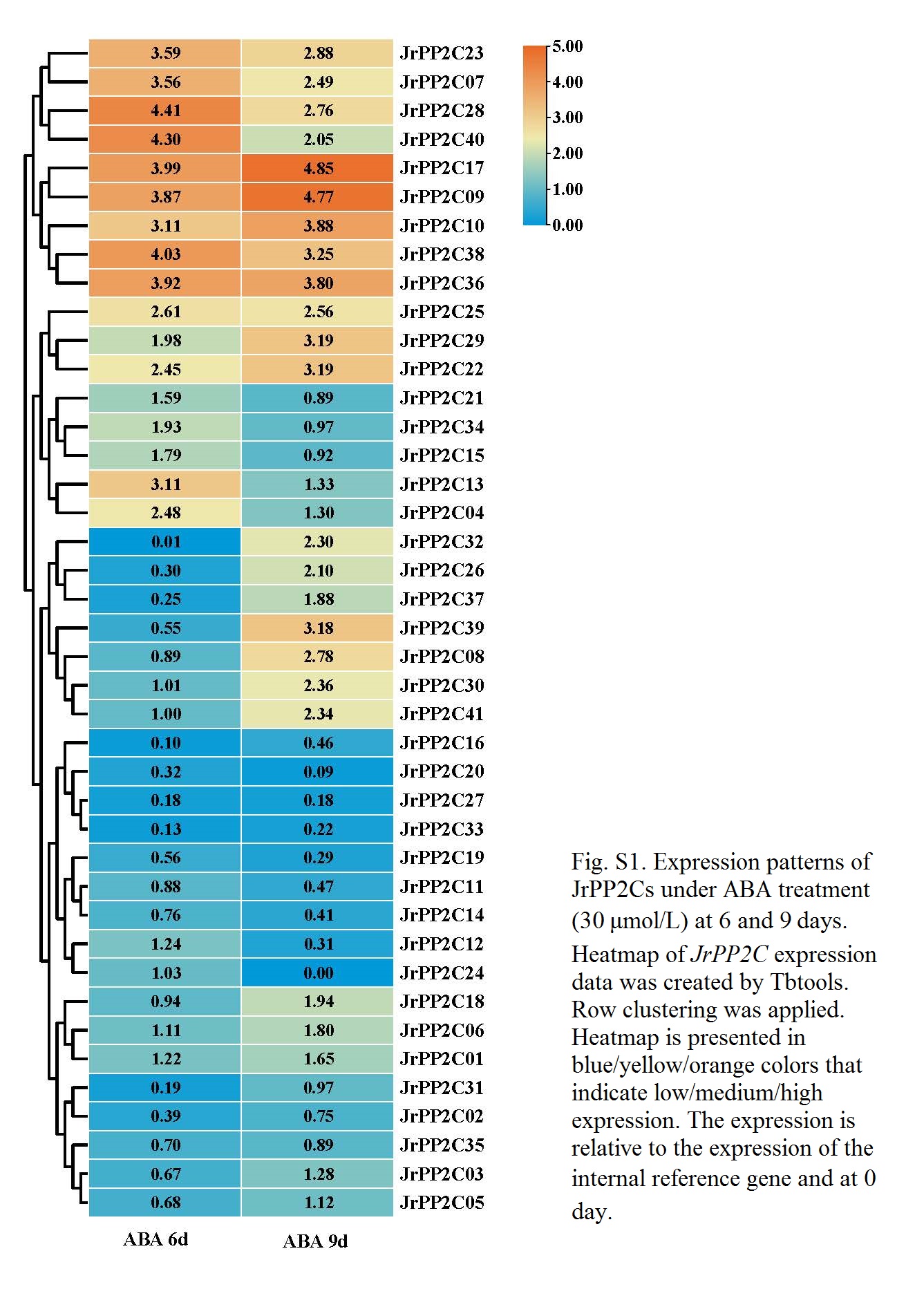

Supplement: Supplementary file 3 — Additional file 3: Fig. S1. Expression patterns of JrPP2Cs under ABA treatment (30 μmol/L) at 6 and 9 days. Heatmap of JrPP2C expression data was created by Tbtools. Row clustering was applied. Heatmap is presented in blue/yellow/orange colors that indicate low/medium/high expression. The expression is relative to the expression of the internal reference gene and at 0 day. [file 12864_2022_8856_MOESM3_ESM.jpg]
